# Variations in suicide method and in suicide occurrence by season and day of the week in Russia and the Nenets Autonomous Okrug, Northwestern Russia: a retrospective population-based mortality study

**DOI:** 10.1186/s12888-015-0601-z

**Published:** 2015-09-23

**Authors:** Yury A. Sumarokov, Tormod Brenn, Alexander V. Kudryavtsev, Odd Nilssen

**Affiliations:** Department of Community Medicine, UiT - The Arctic University of Norway, Tromsø, 9037 Norway; International School of Public Health, Northern State Medical University, Arkhangelsk, Russia

## Abstract

**Background:**

Suicide is an important world health issue, especially in territories inhabited by indigenous people. This investigated differences in suicide rates, suicide methods, and suicide occurrence by month and day of the week among the indigenous and non-indigenous populations of the Nenets Autonomous Okrug (NAO) and to compare the findings from the NAO with national Russian statistics.

**Methods:**

In this retrospective population-based mortality study we investigated all suicides that occurred in the NAO in 2002–2012 (*N* = 252). Suicide method and the month and day of the week suicide occurred was taken from autopsy reports and disaggregated by ethnic group (indigenous and non-indigenous) and sex. Data from the NAO were then compared with national data from the Russian Federal Statistics Service (Rosstat).

**Results:**

Hanging was the most common suicide method in the NAO in both indigenous and non-indigenous populations. The proportion of suicides by hanging among males was lower in the NAO than in national data (69.3 vs 86.2 %), but the inverse was true for females (86.5 vs 74.9 %). Suicide by firearm and by cutting was significantly higher among the indigenous population in the NAO when compared with national data. Peaks in suicide occurrence were observed in May and September in the NAO, whereas national data showed only one peak in May. Suicide occurrence in the indigenous population of the NAO was highest in April, while the non-indigenous population showed peaks in May and September. Suicide occurrence in the NAO was highest on Fridays; in national data this occurrence was highest on Mondays.

**Conclusions:**

We showed different relative frequencies of suicide by hanging, cutting, and firearm, as well as different suicide occurrence by month and day of the week in the NAO compared with Russia as a whole. These results can be used to plan suicide prevention activities in the Russian Arctic.

## Background

Suicide is an important global health issue, particularly in territories inhabited by indigenous people [1]. Indeed, high suicide rates among indigenous people have been reported worldwide since the 1970s [2], with increases observed in Northern Europe, Canada, the United States, Brazil, Australia, New Zealand, and Greenland. An increase in suicide occurrence was registered in Russia in the 1990s [1], with the highest suicide rates seen in Southern Siberia, the Far East, and in the North [3]. During the last decade, the highest suicide rates recorded in Russia were in the Koryak Autonomous Okrug (up to 91.8 per 100,000), the Evenky Autonomous Okrug (up to 121 per 100,000), Chukotka (up to 91.7 per 100,000), and the Nenets Autonomous Okrug (NAO) (up to 119.1 per 100,000), all of which have large indigenous populations [3].

Suicide methods vary by geographic area. Poisoning by pesticide is common in Latin America [4] and in several Asian countries [5, 6], whereas drug poisoning is the most common suicide method in the Nordic countries and the United Kingdom [7, 8]. Suicide by firearm is common in the United States [8], jumping from a height is often used in Hong Kong [6, 8] and Singapore [9], and hanging is common in Eastern Europe and Pakistan [8, 10]. The most violent suicide methods, such as hanging and firearm use, tend to be common in indigenous populations [11–14].

Spring is the season with the highest occurrence of suicides in both the Northern and Southern hemispheres. Several studies from the United States and Canada [15], China [16], Japan [10], Australia [13], South Africa [17], and Europe [18, 19] have reported peaks in suicide occurrence in spring [20]. Variation in suicide occurrence by day of the week has also been reported in different studies, with more suicides occurring on Mondays [21, 22]. A higher suicide occurrence has also been observed in the days following important holidays, especially in men [23].

Our objectives were to investigate the difference in suicide rates, suicide methods, and suicide occurence by month and day of the week among the indigenous and non-indigenous populations of the NAO and to compare the findings from the NAO with national Russian statistics.

## Methods

### Study design and population

**Fig. 1:**
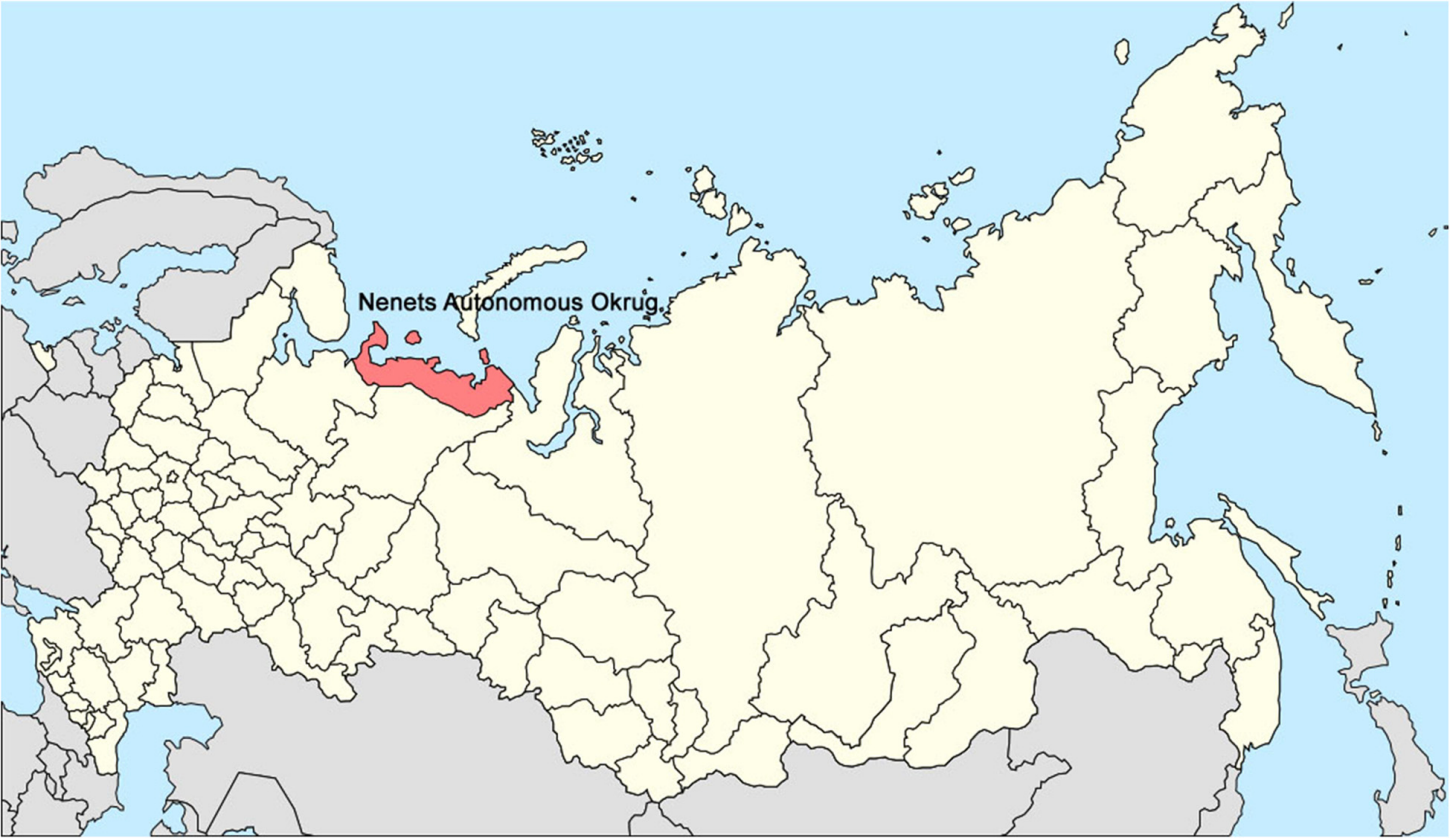
Map of Russia and the Nenets Autonomous Okrug. Source: Wikimedia Commons, Available at: https://commons.wikimedia.org/wiki/File:Map_of_Russia_-_Nenets_Autonomous_Okrug_(2008-03).svg?uselang=ru

The present study is a retrospective population-based mortality study of all suicides that occurred in the NAO and in all of Russia from 1 January 2002 to 31 December 2012 [24]. The NAO is situated in Northwestern Russia, close to the Arctic Ocean (Fig. 1). According to national census data, the total population of the NAO was 41,546 in 2002 and 42,090 in 2010 [25, 26]. The main ethnic groups residing in the NAO in 2010 were Russians (26,648, 63.3 %), Indigenous Nenets (7504, 17.8 %), and Komi (3623, 8.6 %) [26]. The Nenets are one of the largest indigenous populations in Russia. Their traditional lifestyle is nomadic or semi-nomadic, and many Nenets live in temporary (seasonal) settlements. The traditional occupation of Nenets is reindeer herding [27].

### Data sources and description

All cases of suicide (International Classification of Diseases 10^th^ Revision, codes X60-X84 and Y87.0) in the NAO that occurred during the study period were identified through autopsy reports at the Regional Bureau of Forensic Medicine in the city of Naryan-Mar [28]. Data on sex, ethnic group, urban/rural residence, occupation, education level, and marital status for all cases of suicide were obtained as previously described [24]. The distribution of the listed variables have also been reported [24]. Data on the general population of the NAO, such as size and distribution by ethnic group and other considered characteristics (sex, urban/rural residence, occupation, education level, and marital status) were obtained from the Official Russian Censuses of 2002 [25] and 2010 [26]. No other socio-demographic data were available on the NAO population between or after these two Censuses. Nenets ethnicity was established by forensic experts on the basis of family, passport information, and anthropological findings [24]. Subjects with Nenets ethnicity were allocated to the indigenous category, and all others (Russians, Komi, etc.) to the non-indigenous category.

National data on suicides that occurred in the Russian Federation during the study period were obtained from the Russian Federal Statistics Service (Rosstat), using anonymous micro-data on all deaths from external causes recorded in the National Mortality Database in 2002–2012, and disaggregated by sex, suicide method, and month and day of the week suicide occurred.

### Data analyses

We considered the variables ethnic group (indigenous, non-indigenous), sex (male, female), suicide method (hanging, firearm use, cutting, poisoning, jumping from a height, and others), and month and day of the week suicide occurred and compared data from the NAO with national data.

The person-year (PY) approach was used to estimate suicide rates for the NAO and the Russian Federation. Suicide rates were calculated for the period 2002–2012 as number of suicides per 100,000 PYs [24]. The number of PYs of risk in each population was computed as the average population for the NAO and the Russian Federation between the 2002 and 2010 Censuses multiplied by 11 (number of years of observation).

Data are presented as absolute numbers and proportions. The Chi-square test and Fisher’s exact test were used to verify the differences in categorical variables between the studied groups. To test whether the distribution of suicides in the studied groups was equal on the time axes (months of the year and days of the week), the observed distribution of suicides was compared with hypothesized equal distributions by the Chi-square goodness of fit test. Microsoft Excel and IBM SPSS Statistics v.21.0 were used for data storage and analyses.

### Ethical considerations

The study was approved by the Ethical Committee of the Northern State Medical University, Arkhangelsk, Russia.

## Results

Altogether 252 cases of suicide were identified in the NAO during the study period (215 males and 37 females, 67 indigenous and 185 non-indigenous). The suicide rate in the NAO in 2002–2012 was 79.8 per 100,000 PY in the indigenous population and 49.2 per 100,000 PY in the non-indigenous population [24]. Data on all variables considered were available for all cases.

There were 571,162 cases of suicide (474,882 males and 96,280 females) identified in the Russian Federation during the study period. The suicide rate in the Russian Federation in 2002–2012 was 36.0 per 100,000 PY. Data on suicide method were available for all cases; data on the month and day of the week suicide occurred were available for 455,489 (79.7 %) cases.

### Suicide methods

National data showed that hanging was the most frequent suicide method among both males (86.2 %) and females (74.9 %) (Table 1). The proportion suicides by hanging among males in both the indigenous and non-indigenous populations of the NAO (69.3 %) was lower than the proportion observed in national data (*p* <0.001), but among females the proportion of suicides by hanging in both ethnic groups (86.5 %) tended to exceed national estimates (*p* = 0.318). Hanging was the most common suicide method in the indigenous and non-indigenous populations of the NAO and in subgroups of sex (Table 2). When cases of suicide in the NAO were divided by sex only (with no consideration of ethnic group), hanging accounted for higher proportion of suicides among females (86.5 %) than males (69.3 %) (*p* = 0.031). The proportion of suicides by firearm in the NAO was higher than that recorded in national data in both males (20.9 vs 4.7 %) (*p* < 0.001) and females (8.1 vs 0.4 %) (*p* <0.001). In the NAO, indigenous males had a higher relative frequency of suicide by firearm (25.9 %) and cutting (9.3 %) than non-indigenous males (19.3 and 5.6 %, respectively) (*p* >0.05 in both comparisons).

**Table 1:** Suicide methods in the Russian Federation and the NAO by sex (2002–2012)

Suicide method	Russian Federation (*N* = 571,162)			NAO (*N* = 252)		
	Males	Females	Total	Males	Females	Total
Hanging	409,545 (86.2 %)	72,127 (74.9 %)	481,672 (84.3 %)	149 (69.3 %)^a^	32 (86.5 %)	181 (71.8 %)^a^
Firearm use	22,445 (4.7 %)	369 (0.4 %)	22,814 (4.0 %)	45 (20.9 %)^a^	3 (8.1 %)^a^	48 (19.0 %)^a^
Cutting	14,397 (3.0 %)	2120 (2.2 %)	16,517 (2.9 %)	14 (6.5 %)^a^	2 (5.4 %)	16 (6.3 %)^a^
Poisoning	12,344 (2.6 %)	14,031 (14.6 %)	26,375 (4.6 %)	2 (0.9 %)	-	2 (0.8 %)
Jumping from a height	6861 (1.4 %)	4650 (4.8 %)	11,511 (2.0 %)	3 (1.4 %)	-	3 (1.3 %)
Other	9290 (3.1 %)	2983 (3.1 %)	12,273 (2.2 %)	2 (0.9 %)	-	2 (0.8 %)
Total	474,882 (100 %)	96,280 (100 %)	571,162 (100 %)	215 (100 %)	37 (100 %)	252 (100 %)

^a^Different at *p* < 0.05 from the corresponding proportion in the Russian Federation. *NAO* Nenets Autonomous Okrug

**Table 2:** Suicide methods in the NAO by ethnic group and sex (2002–2012)

Suicide method	Non-indigenous population (*N* = 185)			Indigenous population (*N* = 67)		
	Males	Females	Total	Males	Females	Total
Hanging	116 (72 %)	21 (84.6 %)	137 (74.1 %)	33 (61.1 %)	11 (87.5 %)	44 (65.7 %)
Firearm use	31 (19.3 %)	2 (8.3 %)	33 (17.9 %)	14 (25.9 %)	1 (7.7 %)	15 (22.4 %)
Cutting	9 (5.6 %)	1 (4.2 %)	10 (5.4 %)	5 (9.3 %)	1 (7.7 %)	6 (8.9 %)
Poisoning	1 (0.6 %)	-	1 (0.5 %)	1 (1.9 %)	-	1 (1.5 %)
Jumping from a height	3 (1.9 %)	-	3 (1.6 %)	-	-	-
Other	1 (0.6 %)	-	1 (0.5 %)	1 (1.9 %)	-	1 (1.5 %)
Total	161 (100 %)	24 (100 %)	185 (100 %)	54 (100 %)	13 (100 %)	67 (100 %)

*NAO* Nenets Autonomous Okrug

When data from the NAO was compared with national data on suicides, cutting was more frequent suicide method (*p* = 0.003 in total) in the NAO (6.3 % of total cases; 6.5 % in males and 5.4 % in females) than in Russia (2.9 % of total cases; 3.0 % in males and 2.2 % in females). There were no suicides by poisoning in the NAO, whereas suicide by poisoning was the second most frequent suicide method among females (14.6 %) and overall (4.6 %) in Russia (Table 1).

National data showed a higher prevalence of suicide by jumping from a height in females (3^rd^ place at 4.8 %) than males (1.4 %). In the NAO, this method was used only by non-indigenous males (1.4 %) and never by females or indigenous males.

The distribution of suicide methods used by indigenous and non-indigenous females from the NAO was nearly the same (Table 2). Analyses of differences in suicide methods by 10-year age group showed no significant differences between indigenous and non-indigenous populations or within the total population of the NAO (*p* >0.05). Similar analyses were performed to compare suicide methods by socio-demographic subgroup, as defined by urban/rural area of residence, employment category (employers and employees; and other, including unemployed, retirees, students, dependents and the disabled), education level (university/college, secondary school, incomplete secondary, or primary school) and marital status (married, divorced, widowed, and single), but these analyses showed no significant variations (*p* >0.05).

### Variation in suicides by month

**Fig. 2:**
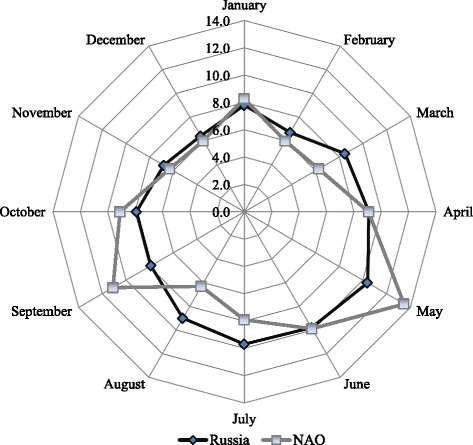
Proportional distribution of suicides in Russia and the NAO by month of the year, 2002–2012 (%). Distribution of suicides in the NAO had significant difference form hypothesized equal distribution (*p* = 0.041). NAO: Nenets Autonomous Okrug

**Fig. 3:**
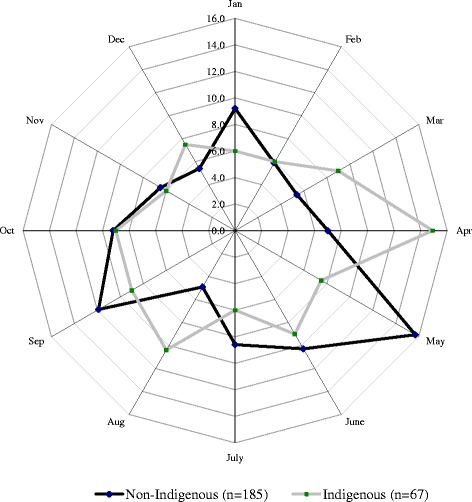
Proportional distribution of suicides in the NAO by month of the year and ethnic group, 2002–2012 (%). Distribution of suicides in the non-indigenous population demonstrate significant difference (*p* = 0.01) from hypothesized equal distribution. In the indigenous population distribution is not significantly different from the equal distribution. NAO: Nenets Autonomous Okrug

Different patterns of suicide occurrence by month were observed in national data and the NAO (Fig. 2). National data demonstrated a tendency toward increased suicide occurrence in spring, with the highest level observed in May (10.4 % of all cases) and the lowest level in December (6.4 %) (Fig. 2). The distribution of suicides in the NAO had two peaks - in May and in September (*p* = 0.041) (Fig. 2). The highest suicide occurrence in the indigenous population was in April (Table 3), whereas the non-indigenous population displayed a suicide peaks in May and September (Fig. 3).

**Table 3:** Suicides in the NAO by ethnic group and month (2002–2012)

Month	Non-indigenous population	Indigenous population	*P*
January	17 (9.2 %)	4 (6.0 %)	0.606
February	11 (5.9 %)	4 (6.0 %)	1.000
March	10 (5.4 %)	6 (9.0 %)	0.307
April	13 (7.0 %)	10 (14.5 %)	0.054
May	29 (15.7 %)	5 (7.5 %)	0.097
June	19 (10.3 %)	6 (9.0 %)	0.758
July	16 (8.6 %)	4 (6.0 %)	0.604
August	9 (4.9 %)	7 (10.4 %)	0.141
September	22 (11.9 %)	6 (9.0 %)	0.512
October	17 (9.2 %)	6 (9.0 %)	0.954
November	12 (6.5 %)	4 (6.0 %)	1.000
December	10 (5.4 %)	5 (7.5 %)	0.552
Total	185 (100 %)	67 (100 %)	

*NAO* Nenets Autonomous Okrug

### Variation in suicides by day of the week

A difference in suicide occurrence by day of the week was observed in the NAO and in national data (Table 4). National data showed an equal distribution of suicides across all days of the week, with a slightly increased number of suicides on Mondays. In the NAO, a lower suicide occurrence was observed on Sundays (*p* = 0.002) and a higher occurrence (18.3 % of all cases) on Fridays (*p* = 0.044). There was no significant difference in the distribution of suicides by day of the week in the indigenous and non-indigenous populations of the NAO (Table 5).

**Table 4:** Suicides in the Russia Federation and in the NAO by day (2002–2012)

Day of the week	Russian Federation	NAO	*P*
Monday	67,799 (14.9 %)	33 (13.1 %)	0.425
Tuesday	65,340 (14.3 %)	34 (13.5 %)	0.699
Wednesday	64,772 (14.2 %)	41 (16.3 %)	0.352
Thursday	63,914 (14.0 %)	36 (14.3 %)	0.908
Friday	63,160 (13.8 %)	46 (18.3 %)	0.044
Saturday	64,118 (14.1 %)	42 (16.7 %)	0.237
Sunday	66,386 (14.6 %)	20 (7.9 %)	0.002
Total	455,489 (100 %)	252 (100 %)	

*NAO* Nenets Autonomous Okrug

**Table 5:** Suicides in the NAO by ethnic group and day (2002–2012)

Day of the week	Non-indigenous population	Indigenous population	*P*
Monday	25 (13.5 %)	8 (11.5 %)	0.744
Tuesday	29 (15.7 %)	5 (7.5 %)	0.099
Wednesday	31 (16.8 %)	10 (14.9 %)	0.728
Thursday	23 (12.4 %)	13 (19.4 %)	0.162
Friday	36 (19.5 %)	10 (14.9 %)	0.410
Saturday	29 (15.7 %)	13 (19.4 %)	0.483
Sunday	12 (6.5 %)	8 (11.9 %)	0.157
Total	185 (100 %)	67 (100 %)	

*NAO* Nenets Autonomous Okrug

## Discussion

This study was conducted to investigate variations in suicide rates, suicide methods, and suicide occurrence by month and by day of the week in the indigenous and non-indigenous populations of the NAO, as well as to compare the findings from the NAO with national Russian statistics. This investigation has been requested by Russian health authorities and its results will be used to plan suicide prevention activities in the Russian Arctic.

### Suicide methods

Our study confirmed previous reports of the high prevalence of suicide by hanging in Russia [3, 29] and showed that hanging was also one of the prevailing suicide methods among the indigenous population. The custom of free-will death among different indigenous populations in the Russian North [30] may explain their use of hanging as a suicide method. According to this custom, the use of strangulation is based on a fear of punishment. In the traditional understanding of the indigenous peoples of the Russian North and Far East, strangulation is considered a “sacrifice to the Gods”. Indeed, according to the spiritual culture of the indigenous Nenets, strangulation blocks the escape route for “bad spirits”, thus protecting those in the community from harm that could be inflicted by these spirits [30]. However, our finding that hanging was less pronounced in the NAO than in Russia at large was a surprise and is a challenge to traditional thinking.

Differences have also been reported in the distribution of suicides by hanging in urban and rural areas in Russia; in Moscow hanging accounts for 60 % of all suicides [29] compared with more than 90 % in rural Tatarstan [31]. The lower proportion of hanging we found in the NAO compared with national data may be explained by the availability of other lethal methods, like firearms and knives used in hunting, especially among men. Indeed, a recent increase in access to weapons in the NAO may explain the increase in suicides by firearm.

For women in the NAO, hanging was used considerably more often than has been reported in other world studies [8]. Western women more often use intoxication with different medicines to commit suicide [8]. Rope is likely more readily available to females in the NAO than medicine, just as firearms are more available to men in the NAO. The same argument could be made for cutting, as possessing a knife is a matter of survival among hunters and reindeer herders. Jumping from a height as a suicide method is less feasible in a society where there are either no heights or only very limited heights. These points should be considered when developing suicide prevention strategies for the NAO. It is impossible to limit the availability of rope, but easy access to firearms can be limited [6, 18].

D. Lester [20] stated that knowledge about suicides in indigenous populations can challenge traditional theories of suicide, and should motivate investigators in the field to take ethnic background into account. The Soviet system, which respected planned economy and efficacy, imposed changes on the Nenets society, the effects of which could be seen at both the individual and the aggregated level [32]. Thus, the high suicide rates we observed in the indigenous population may not be due to religious factors, but instead to an individual reaction to a less predictable society, and the loss of a stable traditional culture [30]. Indeed, this could be one explanation for the newest patterns of suicide in Arctic populations, such as those observed in our study.

### Seasonality of suicides

The analysis of national data from 2002 to 2012 showed an increase of suicides in May and a decrease in November-December. This is in accordance with other studies from Russia [33], which have demonstrated a spring peak and winter decrease in suicides. One may have expected an opposite trend in the NAO, as our study population lives far up in the Arctic where winter depression might be seen as a potential risk factor for suicide. However, the opposite seemed to be true; the peak in suicide occurrence was seen at the end of winter/beginning of spring, when the sunlight starts to return, indicating that spring is the most common period to commit suicide in the NAO.

Similar findings have been reported from other countries [34]. Durkheim pointed out that suicidal behavior is connected to the intensity of circular activities (agricultural) and that longer days increase social stress [35]. Many studies from other countries and areas, such as the United States and Canada [15], Japan [16], Europe [18], Australia [13], and South Africa [17] have demonstrated that suicides are more frequent in spring.

Earlier studies have suggested various reasons for the spring seasonality of suicides, the first one being hormones. Several studies have pointed to increased melatonin, cortisol, serotonin, and L tryptophan activity [36]. These hormones play key roles in emotional regulation, and dysfunctional emotional regulation may lead to depression and consequently to suicide.

Secondly, the social situation in spring combined with a longer working day may have an impact on stress levels and alcohol use, which in turn could lead to psychosocial problems and increased suicide risk. A third reason might be connected with the traditional Nenets lifestyle, which is based on the cycle of the reindeer-herding year. April is usually characterized by the completion of winter reindeer activities. The celebration of Reindeer Herders Day and other festivals at the end of the winter period often bring unforeseen love and sexual activity. Winter is also followed by the movement of families, reindeer, and major changes in life routines, which may increase the risk of suicide. Moreover, recent studies based on mathematical modeling [37] have suggested that sunshine may be a potential driver of suicide. Sunlight can affect the human mind after a long period of little light or darkness. This study lends support to the hypothesis that sunshine up to 10 days prior to suicide may actually facilitate suicide. There is usually a regular increase in sunshine in the spring in the NAO, which may explain the April-May increase in suicide occurrence in both ethnic groups.

### Variations in suicides by day of the week

The distribution of suicides in Russia by day of the week showed a peak on Mondays, which has also been reported in other studies [33]. This is more related to the traditional urban lifestyle, in which weekends and holidays are followed by a higher risk of loneliness, hopelessness, and despair. This post-holiday and Monday suicide risk is in accordance with Durkheim’s theory of “anomia” [22, 35]. In their own traditional environment, the indigenous population in the NAO does not distinguish between weekends and work days, but this is not the case for the non-indigenous population. This difference only influenced the males in our study. For females, the inverse was observed, with indigenous females being more likely to commit suicide on the weekend. Our suggestion is that non-working days and holidays are more risky periods for suicide in indigenous females and non-indigenous males in the NAO.

### Limitations

Many of the observed differences in this paper could only be presented as tendencies of variations. Indeed, although differences were observed, only some of them reached statistical significance. This may be explained by the small absolute number of cases in a sparsely-populated area. The study included all suicides reported in the NAO during the 11-year study period (252 cases), but this sample did not have sufficient statistical power to firmly investigate differences between the indigenous and non-indigenous populations, and specifically in subgroups of these populations defined by other variables in the study.

No information on history of self-harm or suicide attempt was available for the cases of suicide included in the present study. Data for the general population of the NAO were obtained only from the 2002 and 2010 Censuses. There were no other available data on age and sex distribution and other socio-demographic variables in the NAO during the study period. For these reasons the denominators in our calculations of suicide rates were estimates rather than true numbers [24].

### Strengths

The strength of our study is based on the detailed information on suicides from all of Russia, which we compared with data from the NAO. This detailed information from national Russian databases has not been analyzed or published before. Another strength of our study was the active participation of forensic experts. Indeed, autopsy reporting is well organized in the NAO, where planes and helicopters are often used to transport forensic experts to the site of possible suicides [24].

The police and the Investigation Committee of the Russian Federation investigate all suicides in order to identify potential criminal cases, and forensic examination immediately follows the primary police investigation. The role of forensic experts is to classify the cause of death according to the International Classification of Diseases, 10^th^ Revision [1, 28]. The final classification is assigned based on the autopsy and the investigation process, all of which improve the validity of the classification.

To our knowledge, this is the first time that detailed information on suicides from all of Russia has been published. Thus, not only can we present data from the NAO, but we can also compare those data with the rest of Russia with respect to suicide method, and month and day of the week of suicide occurence.

## Conclusions

Hanging was the most common suicide method in both ethnic groups and both sexes in the NAO. The proportion of suicides by hanging in the NAO (both ethnic groups combined) was lower than that among males and higher than that among females in Russia. Suicides by firearm and by cutting in the NAO (in both ethnic groups) were more frequent than in Russia. The proportion of suicides by firearm and by cutting was higher in the indigenous population than the non-indigenous population of the NAO. The occurrence of suicides by month in the NAO differed from national data, which showed a peak in May. The highest suicide occurrence in the indigenous population of the NAO was observed in April. In the non-indigenous population increases were observed in May and September. Suicides occurrence in the NAO was highest on Fridays, whereas in national data it was highest on Mondays. The results of this study may be useful in the planning of prevention activities for different ethnic groups in the Russian Arctic.

## Abbreviations

NAO: Nenets Autonomous Okrug
PY: Person-years
